# Editorial: Latest perspective on microbes detection: from laboratory to on-spot sensor

**DOI:** 10.3389/fmicb.2023.1302805

**Published:** 2023-11-13

**Authors:** Utkarsh Jain, Palmiro Poltronieri, Vincenzina Fusco, Elisabetta Primiceri

**Affiliations:** ^1^School of Health Sciences and Technology, University of Petroleum and Energy Studies, Dehradun, India; ^2^Institute of Sciences of Food Productions (CNR-ISPA), National Research Council, Lecce, Italy; ^3^Institute of Sciences of Food Productions (CNR-ISPA), National Research Council, Bari, Italy; ^4^Department of Physical Sciences and Technologies of Matter, Institute of Nanotechnology, National Research Council (CNR), Lecce, Italy

**Keywords:** on-spot sensors, microbes, gut microbiome, biomarkers, 16S rRNA

It is crucial that we identify new perspectives on microbe detection and front-runners in research and innovation in microbiology. This Research Topic includes innovative investigations conducted by researchers in their specific fields with the objective of spotlighting leading contributions and providing insights on the “*Latest perspectives on microbe detection: from laboratory to on-spot sensor*”, including new, theoretical, experimental, and methodological approaches to challenges and research issues.

Li et al. exemplify the diversity and presence of fungal microbiome in SLE patients through a pilot scale analysis. Sixty-four subjects (19 SLE, 20 RA, 9 UCTD, and 16 HC) were chosen for the experiment and fecal samples from all were collected. ITS sequencing and 16S rRNA gene sequencing were employed for the detection of gut fungi and bacteria and the alpha and beta diversities of the microbial community were examined. Among SLE, RA, UCTD, and HC various gut fungi with different abundance patterns taxa were identified. Moreover, the symbiosis of bacteria and fungi in the gut of SLE patients showed an alteration compared to the HCs.

Cha et al. profiled the gut microbiome of neonates using two different sequencing methods. This study focuses on the assessment of variation in gut microbiome in premature and full-term infants using an advanced third-generation technique known as Oxford Nanopore Technology and the results were compared with a second-generation sequencing technique, Illumina. In total, 69 fecal samples were collected and, by employing 16S rRNA gene sequencing using Nanopore technology, the profiling of gut microbiota colonization was conducted. Bioinformatic analysis showcased the difference in the above two infants. Both the infants were dominated by *Staphylococcus* and *Enterococcus*, whereas in preterm infants, *Klebsiella*, a pathogenic bacterium, was found to be dominant as compared to *Lactobacillus* and *Streptococcus* in term infants. This study showed a high correlation between the Nanopore Sequencing Technology and Illumina Sequencing technology. Thus, in clinical settings, this Nanopore sequencing has the potential to detect pathogens in neonates.

Zhang et al. exemplified various studies and concluded that Colorectal Cancer (CRC), which is among the most severe and common threats, can be treated in its early stage as in most cases it arises from colorectal polyps. For this to be performed, a diagnosis method compliant with people that is non-invasive and inexpensive is needed. Salivary and fecal microbiota were suggested as biomarkers for predicting colorectal polyps. The authors performed the detection of the distribution of microbial communities in salivary and fecal samples from the Colorectal Polyp Group (CP) and Healthy Control Group (HC) by full-length 16S rRNA sequencing and by using various statistical tools such as Linear Discriminant Analysis Effect Size (LEfSe), ROC Curve, etc. evaluating if the diversity found could be a potential biomarker for diagnosis. They also established a significant difference between the increased diversity of deleterious bacteria and the decreased diversity of advantageous bacteria between CP and HC groups.

Cai et al. investigated the consequences of oxygen toxicity on the gut microbiota and metabolome by comparing the metagenomic sequencing study of fecal samples of two mice groups: the control group was provided with 21% Fractional inspired oxygen and the oxy-group was provided with 80% Fractional inspired oxygen. By employing Linear Discriminant Analysis Effect Size (LEfSe), it was concluded that the difference in microbial diversity is significant and *Firmicutes* are the biomarker for the oxy group whereas *Muribaculaceae* and its isolate-37 are the biomarkers for the control group. Carbohydrate and lipid metabolism were affected by hyperoxia. HPLC-MS analysis of a fecal sample with blood for the metabolomic study was also performed, concluding that it affected significantly the production of certain metabolites such as less production of 11-dehydro Thromboxane B2-d4, thus diminishing the metabolism of linoleic and alpha-linolenic acid and inhibiting a serum metabolite, 1-docosanoyl-glycero-3-phosphate. This also suppressed the hypoxia Inducible Factor-1 and Glucagon Signaling pathways.

Asare et al. worked for the rapid identification of human commensal intestinal bacteria of class *Clostridia*. This study aimed to generate a database of MALDI-TOF MS plugins that could be helpful in the rapid identification of any non-pathogenic, commensal bacteria present in the human gut. From 142 bacterial strains (present within the class *Clostridia*), a Mass Spectral Profile (MSP) database was created. The database was validated using 58 full-length 16S rRNA sequence isolates taken from two different laboratories. The database identified 98% and 93% strains when the Bruker database was combined with this MS plugin. Moreover, the combination of this plugin with the original library identified 80% of the unknown strains, which were earlier identified as 60% diversity.

Liu et al. reviewed some studies on Eucommia ulmoides Oliver (EuO), a natural medicine helpful in improving intestinal flora composition in fishes. The authors examined changes in the intestinal structure and flora of fish, before and after the supplementation with EuO, against the disorders caused by the high starch diet, and also performed the analysis of its effects on the functionality of the immune and digestive system. Moreover, they recorded the effects of EuO supplementation on *Micropterus salmoides*.

In recent years, a wide emphasis has been given to microbial detection as these “miniature creatures” are responsible for many beneficial and harmful interactions within the host. As a result, this Research Topic encompasses original studies, offering insights into microbial detection, spanning from laboratory techniques to real-time sensor applications, thus making microbial detection an easy and effective process. This Research Topic of “*Latest perspectives on microbe detection: from laboratory to on-spot sensor*” intends to inspire new researchers to work in this research area in the hope that their work to lead to new innovations in the future.

## Author contributions

UJ: Writing—original draft, Writing—review & editing. PP: Writing—original draft, Writing—review & editing. VF: Writing—original draft, Writing—review & editing. EP: Writing—original draft, Writing—review & editing.

